# Multimodal integration of radiology, pathology and genomics for prediction of response to PD-(L)1 blockade in patients with non-small cell lung cancer

**DOI:** 10.1038/s43018-022-00416-8

**Published:** 2022-08-29

**Authors:** Rami S. Vanguri, Jia Luo, Andrew T. Aukerman, Jacklynn V. Egger, Christopher J. Fong, Natally Horvat, Andrew Pagano, Jose de Arimateia Batista Araujo-Filho, Luke Geneslaw, Hira Rizvi, Ramon Sosa, Kevin M. Boehm, Soo-Ryum Yang, Francis M. Bodd, Katia Ventura, Travis J. Hollmann, Michelle S. Ginsberg, Jianjiong Gao, MSK MIND Consortium, Rami Vanguri, Matthew D. Hellmann, Jennifer L. Sauter, Sohrab P. Shah

**Affiliations:** 1 Computational Oncology, Department of Epidemiology and Biostatistics, Memorial Sloan Kettering Cancer Center, New York, NY USA; 2 Thoracic Oncology Service, Division of Solid Tumor Oncology, Department of Medicine, Memorial Sloan Kettering Cancer Center, New York, NY USA; 3 Druckenmiller Center for Lung Cancer Research, Memorial Sloan Kettering Cancer Center, New York, NY USA; 4 Department of Radiology, Memorial Sloan Kettering Cancer Center, New York, NY USA; 5 Department of Pathology and Laboratory Medicine, Memorial Sloan Kettering Cancer Center, New York, NY USA; 6 Weill Cornell/Rockefeller/Sloan Kettering Tri-Institutional MD-PhD Program, New York, NY USA; 7 Parker Institute for Cancer Immunotherapy, San Francisco, CA USA; 8 Kravis Center for Molecular Oncology, Memorial Sloan Kettering Cancer Center, New York, NY USA

**Keywords:** Non-small-cell lung cancer, Tumour biomarkers, Cancer, Machine learning

## Abstract

Immunotherapy is used to treat almost all patients with advanced non-small cell lung cancer (NSCLC); however, identifying robust predictive biomarkers remains challenging. Here we show the predictive capacity of integrating medical imaging, histopathologic and genomic features to predict immunotherapy response using a cohort of 247 patients with advanced NSCLC with multimodal baseline data obtained during diagnostic clinical workup, including computed tomography scan images, digitized programmed death ligand-1 immunohistochemistry slides and known outcomes to immunotherapy. Using domain expert annotations, we developed a computational workflow to extract patient-level features and used a machine-learning approach to integrate multimodal features into a risk prediction model. Our multimodal model (area under the curve (AUC) = 0.80, 95% confidence interval (CI) 0.74–0.86) outperformed unimodal measures, including tumor mutational burden (AUC = 0.61, 95% CI 0.52–0.70) and programmed death ligand-1 immunohistochemistry score (AUC = 0.73, 95% CI 0.65–0.81). Our study therefore provides a quantitative rationale for using multimodal features to improve prediction of immunotherapy response in patients with NSCLC using expert-guided machine learning.

## Abstract (web summary)

Shah and colleagues used machine learning to integrate features from computed tomography scan images, programmed death ligand-1 (PD-L1) immunohistochemistry and genomics into a multimodal predictor of response to anti-PD(L)1 in patients with NSCLC that outperformed single features such as tumor mutational burden.

## Main

Immunotherapies blocking programmed cell death protein 1 (PD-1) and its ligand (PD-L1)^1^ to activate and reinvigorate cytotoxic antitumor T cells^2^ have rapidly altered the treatment landscape of NSCLC^3–8^. In just 4 years, PD-1/PD-L1 pathway blockade (abbreviated as PD-(L)1) therapy has become a routine component of treatment for nearly all patients and is now being tested in earlier stages of lung cancer and in combination with other therapies^9,10^. These treatments represent a potential for long-term, durable benefit for a subset of individuals with advanced lung cancer^11,12^. Consequently, there is a need to develop predictive biomarkers.

Multiple independent analyses have pinpointed individual baseline clinical features as potential independent predictors of response (such as antibiotic use^13^, systemic steroid use^14^ and neutrophil-to-lymphocyte ratio at diagnosis^15^), individual genomic alterations (including mutations in *EGFR*^16^ and *STK11* (ref. ^17^)) and densities of intratumoral cytotoxic T-cell populations^18–20^. Currently there are only two US Food and Drug Administration (FDA)-approved predictive biomarkers for immunotherapy in NSCLC: tumor PD-L1 expression assessed by immunohistochemistry (IHC)^21^ and tumor mutation burden (TMB)^22–24^; however, they are only modestly helpful. For example, PD-L1 expression only modestly distinguished long-term response in the 5-year overall survival (OS) report of Keynote-001 (ref. ^12^).

In contrast to previous approaches^25^, we sought to develop a model that integrates and synthesizes multimodal data routinely obtained during clinical care to predict response to immunotherapy. Patients diagnosed with advanced NSCLC undergo standard-of-care tests, which generate valuable data such as PD-L1 expression patterns in diagnostic tumor biopsies^26^ and radiological computed tomography (CT) images used in the staging of lung cancer^27^. The raw data from these modalities are amenable to automated feature extraction with machine learning and image analysis tools. Accordingly, machine-learning-based integration of these modalities represents an opportunity to advance precision oncology for PD-(L)1 blockade by computing patient specific risk scores^28^. Previous work on automated deep-learning methods to predict immunotherapy outcomes from CT scans has shown predictive capacity from specific lesion types^29^. One previous study uses CT scans, laboratory data and clinical data to predict NSCLC immunotherapy outcomes, but incorporates only *EGFR* and *KRAS* mutational status^30^. However, in general, the relative predictive capacity of unimodal histology, radiology, genomic and clinical features compared to an integrated model remains poorly understood. This is in part due to a lack of datasets with multiple modalities available from the same set of patients from which systematic evaluation can be undertaken. Here, we present a multidisciplinary study on a rigorously curated multimodal cohort of 247 patients with NSCLC treated with PD-(L)1 blockade to develop a dynamic deep attention-based multiple-instance learning model with masking (DyAM), to predict immunotherapy response. We present a quantitative evaluation and predictive capacity of an adaptively weighted multimodal approach relative to the unimodal features derived from histology, radiology, genomics and standard-of-care approved biomarkers.

## Results

### Clinical characteristics of patients with NSCLC who received PD-(L)1 blockade

**Fig. 1:**
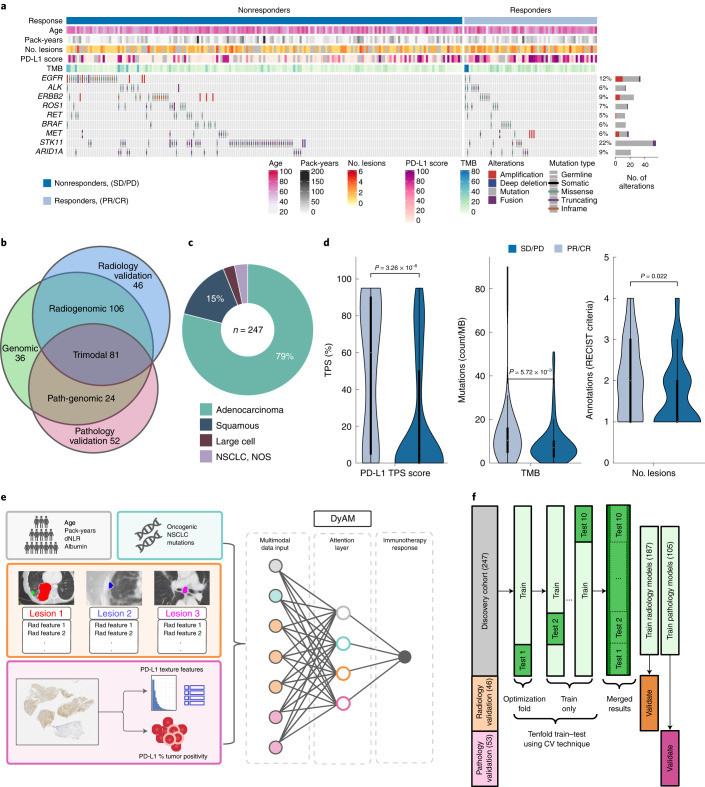
Multimodal cohort characteristics and schema outlining the project. **a**, Multimodal cohort heat map listing clinical, pathological, radiomic and genomic characteristics for *n* = 247 patients. The stacked bar plot shows the number of patients with a genomic alteration, where the colors correspond to alteration types. **b**, Cohort modality overview Venn diagram, with the number of patients exclusively in each category. **c**, Lung cancer histology breakdown. **d**, Distribution of PD-L1 TPS (*n* = 201 patients), TMB (*n* = 247 patients) and number of annotated lesions (*n* = 187 patients) between responders (PR/CR) and nonresponders (SD/PD). The interior box-and-whisker bars show the mean as a white dot, the IQR (25–75%) as a black bar and the minimum and maximum as whiskers up to 1.5 × IQR. *P* values were obtained from a two-sided Mann–Whitney–Wilcoxon test. **e**, Analysis overview using DyAM to integrate multiple modalities to predict immunotherapy response. **f**, Train–test–validate, breakdown and optimization scheme. CV, cross-validation. Source data

We identified 247 patients at Memorial Sloan Kettering (MSK) Cancer Center with advanced NSCLC who received PD-(L)1-blockade-based therapy with baseline data and known outcomes between 2014 and 2019 (cohort characteristics are shown in Table 1 and Fig. 1a–c), referred to as the multimodal cohort. As only 25% of the cohort responded to immunotherapy (consistent with real-world proportions), we consistently use class-balancing in our predictive models.

**Table 1:** Patient characteristics of the three orthogonal cohorts used in this study: multimodal, radiology and pathology

Characteristics	Multimodal	Radiology	Pathology
	(*n* = 247)	(*n* = 50)	(*n* = 52)
	*n* (%)	*n* (%)	*n* (%)
**Age, median (range)**	68 (38–93)	67 (45–86)	71 (30–89)
**Sex**			
Male	113 (46)	24 (48)	23 (44)
Female	134 (54)	26 (52)	29 (56)
**Performance status**			
ECOG 0/1	222 (90)	25 (50)	49 (94)
ECOG ≥2	25 (10)	25 (50)	3 (6)
**Smoking status**			
Current/former	218 (88)	38 (76)	47 (90)
Never	29 (12)	12 (24)	5 (10)
**Histology**			
Adenocarcinoma	195 (79)	39 (78)	32 (62)
Squamous	37 (15)	8 (16)	11 (21)
Large cell	7 (3)	2 (4)	0 (0)
NSCLC, NOS	8 (3)	1 (2)	9 (17)
**Line of therapy**			
1	78 (32)	5 (10)	35 (67)
2	136 (55)	24 (48)	11 (21)
≥3	33 (13)	21 (42)	6 (12)
**Therapy type**			
Anti-PD-(L)1 monotherapy	235 (95)	48 (96)	52 (100)
Anti-PD-(L)1 + CTLA-4 combination	12 (5)	2 (4)	0 (0)
**PD-L1 expression**			
0	114 (46)	–	0 (0)
1–49%	51 (21)	–	13 (25)
≥50%	82 (33)	–	39 (75)
**Tissue site**		–	
Lung	109 (44)	–	25 (48)
Pleura/pleural fluid	19 (8)	–	1 (2)
Lymph node	45 (18)	–	10 (19)
Liver	11 (4)	–	2 (4)
Bone	16 (7)	–	3 (6)
Adrenal	11 (4)	–	1 (2)
Other	36 (15)		10 (19)
**TMB**			
≥10 mutations per Mb	155 (63)	9 (29)	22 (58)
<10 mutations per Mb	92 (37)	22 (71)	16 (42)
**Best overall response**			
CR/PR	62 (25)	11 (22)	14 (27)
SD/PD	185 (75)	39 (78)	38 (73)

ECOG, Eastern Cooperative Oncology Group; NOS, not otherwise specified.

Best overall response to PD-(L)1 blockade was retrospectively assessed by thoracic radiologists with RECIST (v.1.1) criteria resulting in 137 (55%) patients with progressive disease (PD), 48 (20%) with stable disease (SD), 56 (23%) with partial response (PR) and 6 (2%) with complete response (CR). In this analysis, the cohort was binarized as responders (CR/PR) and nonresponders (SD/PD), resulting in median progression-free survival (PFS) and OS of 2.7 months (95% CI 2.5–3.0) and 11.4 months (95% CI 10.3–12.8), respectively.

Two additional cohorts were assembled to validate unimodal features extracted from radiological and histological data, referred to as the radiology (*n* = 50) and pathology (*n* = 52) cohorts, respectively (Table 1). Patients in these two cohorts did not meet the inclusion criteria for the multimodal cohort due to missing data from one of the other modalities.

### Establishing an NSCLC multimodal cohort to predict response

Standard clinical biomarkers, including PD-L1 tumor proportion score (TPS) and TMB were significantly different between responders and nonresponders in the multimodal cohort; however, classification models using these features were unable to completely separate the two groups (TPS, AUC = 0.73, 95% CI 0.65–0.81; TMB, AUC = 0.61, 95% CI 0.52–0.69; Fig. 1d). Thus, we assembled routinely collected clinical information, CT scans, digitized PD-L1 IHC in tissue containing NSCLC and genomic features from the MSK-IMPACT clinical sequencing platform^31^ for the multimodal cohort. We used these data to establish a multimodal biomarker. We first quantified the predictive capacity of each modality individually, before assembling all available data into a multimodal biomarker to build an algorithm predictive of response (Fig. 1e). We performed tenfold cross-validation to obtain model predictions for the entire multimodal cohort by merging results from the test sets (Fig. 1f).

### CT features only modestly separate responders

**Fig. 2:**
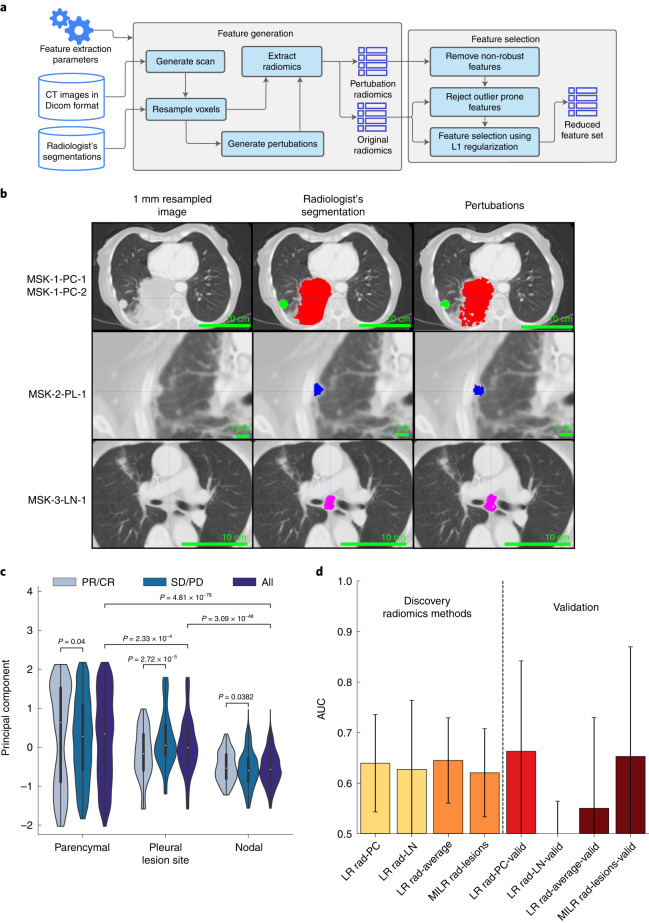
Extraction of CT radiomics features and association with response. **a**, Radiomics feature extraction pipeline using expert segmented thoracic CT scans. Superpixel-based perturbations on original segmentations used for feature selection. **b**, Three expert CT segmentation examples including lung parenchymal (PC) (top), pleural (PL) (middle) and lymph node (LN) lesions (bottom) representative of *n* = 187 patients, with the original image, segmentation and randomized contour example. **c**, Principal-component decomposition distribution of radiomics features for superpixel-based perturbations across tissue sites. The interior box-and-whisker bars show the mean as a white dot, the IQR (25–75%) as a black bar and the minimum and maximum as whiskers up to 1.5 × IQR. *P* values were obtained from the two-sided Mann–Whitney–Wilcoxon test for *n* = 2,040 PC perturbations with *n* = 540 in the responding group and *n* = 1,500 in the nonresponding group; *n* = 330 PL perturbations with *n* = 130 in the responding group and *n* = 200 in the nonresponding group; and *n* = 960 LN perturbations with *n* = 360 in the responding group and *n* = 600 in the nonresponding group. **d**, Response prediction performance using LR classifiers for each type of lesion as well as averaging-based patient-level prediction aggregation by averaging (LR Rad-Average) outcomes across all lesions and the multiple-instance learning model. Results with AUC < 0.5 are not shown. The bar height and error bar represent the AUC and associated 95% CI based on DeLong’s method^51^ for *n* = 187 and *n* = 46 patients in the multimodal and validation cohorts, respectively. Source data

Of 247 patients, 187 (76%) had disease that was clearly delineated and separable from adjacent organs. These 187 patients included 163 (87%) with lung parenchymal lesions, 21 (11%) with pleural lesions and 67 (36%) with pathologically enlarged lymph nodes. For each patient, up to six lesions were segmented and site-annotated by three board-certified thoracic radiologists (N.H., A.P. and J.A-F.). To ensure consistency in CT protocols, our analysis was limited to chest imaging with contrast. The mean segmented volume for lung parenchymal, pleural and nodal lesions was 24 (range 0.14–50, interquartile range (IQR) 15–48), 12 (range 0.31–209, IQR 16–26) and 9.4 (range 0.82–42, IQR 37–67) cm^3^, respectively. We extracted robust features from the original radiologist segmentations that were augmented by superpixel-based perturbations (Fig. 2a,b). Principal-component analysis (PCA) of all radiomics features of the original and perturbed segmentations (Fig. 2c) showed lesion-wise similarity, indicating broad preservation of the underlying texture and significant differences in the principal component by lesion type. The similarity of radiomics features by lesion type across patients motivated building site-specific classification models. L1-regularized logistic regression (LR) models selected an average of 35, 10 and 25 features from lung parenchymal, pleural and nodal lesions, respectively, which we used for downstream prediction of immunotherapy response (Fig. 2d). The logistic model built from features derived from pleural nodules alone was unsuccessful outside of training data (AUC = 0.28, 95% CI 0.04–0.52) compared to lung parenchymal nodules (AUC = 0.64, 95% CI 0.54–0.74) and pathologically enlarged lymph nodes (AUC = 0.63, 95% CI 0.49–0.77). The model based on enlarged lymph nodes did not consistently converge, with over 20% of models performing worse than random chance with repeated subsampling. We aggregated the average individual lesion predictions to construct patient-level response predictions, which resulted in an overall AUC = 0.65 and 95% CI 0.57–0.73. We also developed an alternative model that analyzed all lesions from each patient without separation into categories using multiple-instance learning, which resulted in similar, albeit lower, performance (AUC = 0.61, 95% CI 0.52–0.70).

CT-based predictions of response were validated in the radiology cohort, consisting of 50 patients (Table 1) with expert segmentation, resulting in 40 lung parenchymal lesions, 8 pleural lesions and 22 enlarged lymph nodes. The predictive ability from features extracted from the lung parenchymal lesions (AUC = 0.66, 95% CI 0.48–0.84) was consistent with the multimodal cohort (AUC = 0.64, 95% CI 0.54–0.74), as were the averaging (AUC = 0.55, 95% CI 0.37–0.73) and multiple-instance learning-based aggregation models (AUC = 0.65, 95% CI 0.44–0.87). Taken together, discriminating clinical end points by CT-derived features was modest and primarily driven by texture in the lung parenchymal lesions; however, lesion-specific feature extractions were propagated for use in the multimodal model, where relative contributions to predictive capacity were evaluated.

### PD-L1 texture features approximate pathologist assessments

**Fig. 3:**
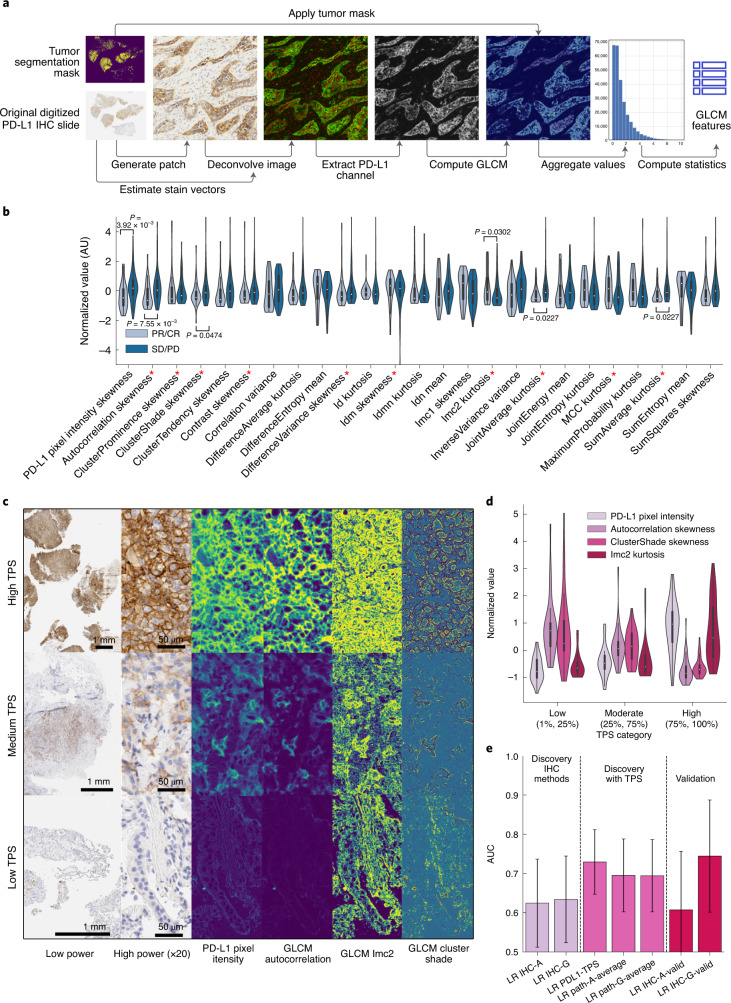
PD-L1 immunohistochemistry feature derivation and prediction of response. **a**, Analysis pipeline to extract image-based IHC texture starting from scanned PD-L1 IHC slides. **b**, Normalized-value distributions of GLCM and pixel intensity-derived image features stratified by response for the best performing summary statistic in each GLCM class. Features indicated by the red asterisk emerged as salient features in the LR fit with *n* = 42 responders and *n* = 63 nonresponders. **c**, Three representative PD-L1 IHC slides from *n* = 105 samples corresponding to the maximum (top), median (middle) and minimum (bottom) of the GLCM autocorrelation distribution, with low power, high power, stain intensity and pixel-wise GLCM sample patches. **d**, Correspondence of the example GLCM features in **c** between low, medium and high bins of TPS. The interior box-and-whisker bars show the mean as a white dot, the IQR (25–75%) as a black bar and the minimum and maximum as whiskers up to 1.5 × IQR for *n* = 105 samples. **e**, Response prediction performance using LR classifiers with PD-L1 features including TPS (LR PD-L1-TPS), pixel and GLCM autocorrelation image features (LR IHC-A), only the complete GLCM features (LR IHC-G) and the result of aggregating patient-level predictions by averaging classifier outcomes from LR IHC-A, IHC-G and LR PD-L1-TPS (LR Path-Average). The bar height and error bar represent the merged AUC and associated 95% CI based on DeLong’s method^51^ for *n* = 105 and *n* = 52 patients in the multimodal and validation cohorts, respectively. Source data

**Extended Data Fig. 1:**
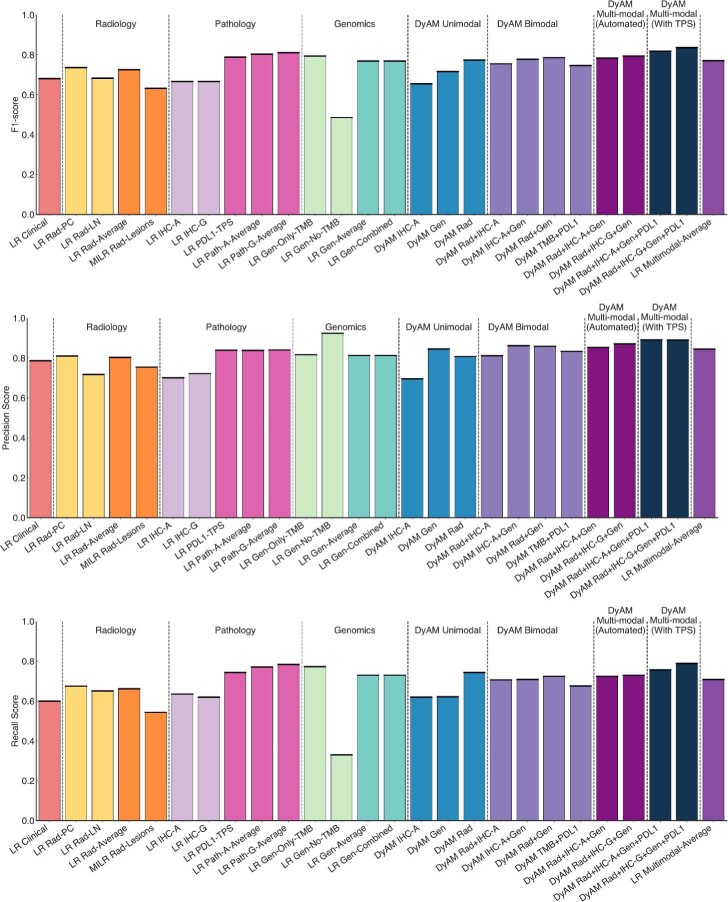
F1, precision and recall score summary plot. The F1-score (top), precision score (middle) and recall score (bottom) for all models tested. The bar heigh represents the metric calculated on the merged classifier results across the entire cohort with N = 247 patients. Source data

We next studied digitized pre-treatment PD-L1 IHC performed on tumor specimens meeting quality control standards (*n* = 201 patients (81%)). A total of 105 (52%) tumor slides showed positive PD-L1 IHC staining (TPS ≥ 1%) and were used to extract IHC texture, a characterization of PD-L1 IHC based on the spatial distribution of expression (Fig. 3a). IHC texture was composed of features with a wide range of statistical association to immunotherapy response (Fig. 3b). The most predictive feature, skewness of the gray-level co-occurrence matrix (GLCM) autocorrelation matrix, correlated with both TPS and PFS (AUC = 0.68, *r*^2^_TPS_ = −0.38, *r*^2^_PFS_ = 0.17, *n* = 105). The maximum, median and minimum of autocorrelation (a measure of the coarseness of the texture of an image) skewness corresponded broadly with PD-L1 IHC stain intensity as well as with contrast and edges in PD-L1 intensity between cells (Fig. 3c). Additional significant features from the LR fit included cluster shade skewness and Imc2 kurtosis, which were less sensitive to the overall PD-L1 stain intensity (Fig. 3c). Furthermore, distributions of GLCM autocorrelation were significantly and inversely associated with pathologist-assessed PD-L1 TPS (Fig. 3d) indicating automated feature extraction with IHC texture could approximate expert thoracic pathologist assessment. LR modeling using 18 features based on the autocorrelation matrix and statistics of the pixel intensity distribution (IHC-A) resulted in prediction accuracy of AUC = 0.62 (95% CI 0.51–0.73), which was comparable to lesion-wide radiological averaging (AUC = 0.65, 95% CI 0.57–0.73), but inferior to the pathologist-assessed PD-L1 TPS (AUC = 0.73, 95% CI 0.65–0.81) (Fig. 3e). While including TPS and IHC-A features reduced the AUC (Fig. 3e), other classifier performance metrics including accuracy, recall and F1-score increased (Extended Data Fig. 1 and Supplementary Table 1). Including all 150 GLCM features (IHC-G) resulted in a prediction accuracy of AUC = 0.63 (95% CI 0.52–0.74).

Using IHC-A features, we validated our findings in the pathology cohort, which consisted of 52 patients with positive PD-L1 expression. The result was consistent with the multimodal cohort (multimodal cohort, AUC = 0.61, 95% CI 0.46–0.76; versus pathology cohort, AUC = 0.62, 95% CI 0.51–0.73). GLCM autocorrelation (Fig. 3c) mean (multimodal cohort, AUC = 0.67, 95% CI 0.56–0.78; versus pathology cohort, AUC = 0.72, 95% CI 0.58–0.86) and skewness (multimodal cohort, AUC = 0.69, 95% CI 0.58–0.80; versus pathology cohort, AUC = 0.74, 95% CI 0.60–0.88) were also consistent with the multimodal cohort. IHC-G features had higher performance in the pathology cohort with AUC = 0.74 (95% CI 0.67–0.81).

### Genomic predictors of response from clinical sequencing data

**Fig. 4:**
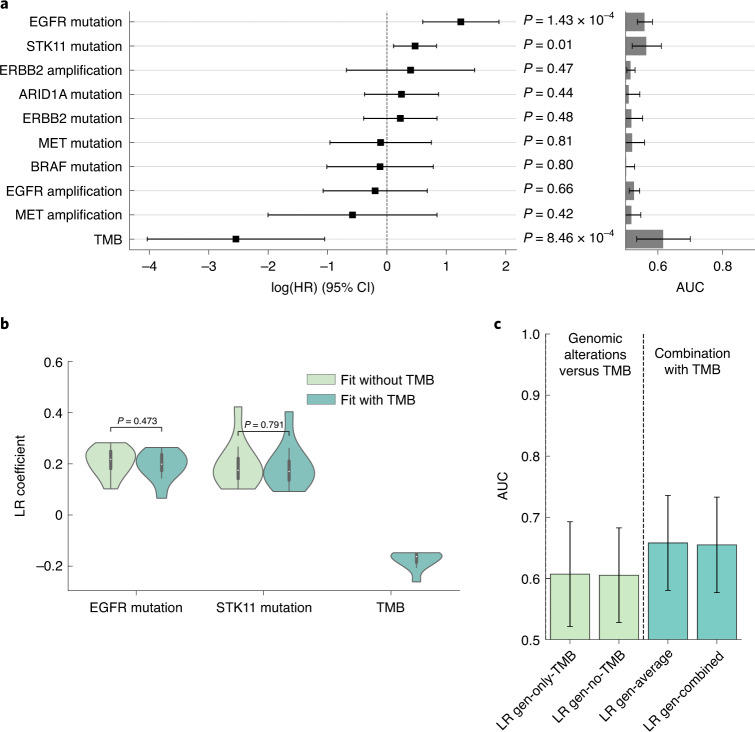
Modeling of response from genomic alterations and TMB. **a**, aHRs using Cox proportional hazard model analysis of genomic variables alongside single feature AUCs. *P* values were obtained from the likelihood-ratio test for *n* = 247 patients. **b**, Comparison of EGFR and STK11 feature coefficients with and without the inclusion of TMB in the model for *n* = 10 fits. The interior box-and-whisker bars show the mean as a white dot, the IQR (25–75%) as a black bar and the minimum and maximum as whiskers up to 1.5 × IQR. **c**, AUCs resulting from models using only TMB, genomic alterations (without TMB), averaging predictions from the TMB and alterations models and fitting a model with both TMB and genomic alterations. The bar height and error bar represent the AUC and associated 95% CI based on DeLong’s method^51^ for *n* = 247 patients with genomic data. Source data

We then assessed features derived from clinical sequencing data from MSK IMPACT^35^, a 341–468 gene targeted next-generation sequencing assay performed on formalin-fixed paraffin-embedded (FFPE) tumor tissue along with matched healthy specimens (blood) from each patient to detect somatic gene alterations with a broad panel. Using multivariate analysis on PFS in the multimodal cohort, alterations of *EGFR* (*n* = 22/247, 8.9%; adjusted hazard ratio (aHR) = 2.14, 95% CI 1.06–4.31, *P* = 0.03), *STK11* (*n* = 44/247, 17.8%; aHR = 2.53, 95% CI 1.71–3.74, *P* < 0.005) and tumor mutation burden (TMB) (median 7 mt per mb, range 0–90; aHR = 0.14, 95% CI 0.02–0.88, *P* = 0.04) exhibited significant aHR in a multivariable analysis of mutated oncogenes (*EGFR*, *ALK*, *ROS1*, *RET*, *MET*, *ERBB2* and *BRAF*), tumor suppressor genes (*STK11*), transcription regulator (*ARID1A*) and TMB (Fig. 4a). LR was used to determine the association between TMB and response (AUC = 0.61, 95% CI 0.52–0.70). The predictive ability of genomic alterations commonly studied in NSCLC excluding TMB (AUC = 0.61, 95% CI 0.53–0.69) was inferior to the model trained using TMB and genomic alterations (AUC = 0.65, 95% CI 0.60–0.80); however, the model performed similarly using the average of TMB and genomic alterations (AUC = 0.65) (Fig. 4c). These features were independent predictors; *EGFR* and TMB were uncorrelated (*r* = −0.03, 95% CI −0.15–0.09) as well as STK11 and TMB (*r* = −0.01, 95% CI −0.14–0.11) and inclusion of TMB had no impact on the coefficients of *EGFR* and *STK11* in the fit (Fig. 4b). These results were broadly consistent with previous reports^25^, establishing their suitability in this cohort for multimodal data integration.

### Multimodal integration via deep-learning improves prediction

**Fig. 5:**
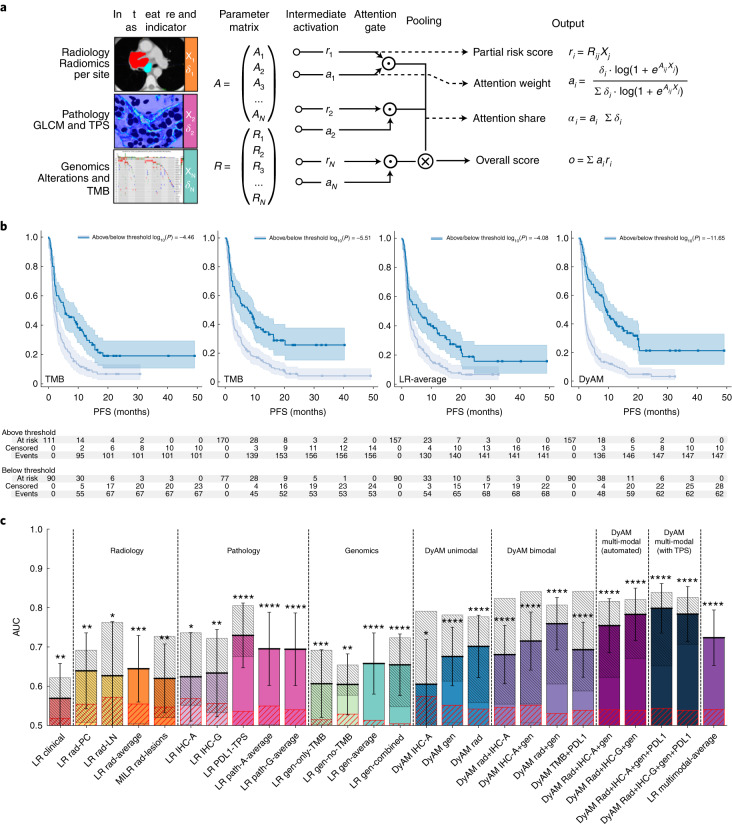
DyAM-based unimodal and multimodal prediction of response. **a**, DyAM was used for multimodal integration. CT segmentation-derived features were separated by lesion type (lung PC, PL and LN) with separate attention weights applied. Attention weights are also used for genomics and PD-L1 IHC-derived features to result in a final prediction of response. The model’s modality specific risk score, attention scores and overall score can be analyzed. Mets, metastases. **b**, Overall score analysis: Kaplan–Meier survival analysis using DyAM to integrate all three modes of data results in significant separation of responders from nonresponders. *P* values were obtained from the log-rank test. **c**, Response predictions summary plot with combinations of input data modalities using DyAM and LR models. The coarse, red-hatched regions represent the 1-sigma error on the permutation-tested AUC measurement and the fine, gray-hatched regions represent the 1-sigma error from repeated subsampling. The bar height and error bar represent the AUC and associated 95% CI based on DeLong’s method^51^. For *n* = 247 patients for the clinical result, *n* = 187 for the radiology results, *n* = 105 for the pathology results, *n* = 247 for the genomic results and *n* = 247 for the multimodal LR and DyAM results. The asterisks indicate the number of s.d. between the merged AUC and the permutation-tested AUC, with 1–4 asterisks representing 1–4 + s.d. Source data

**Extended Data Fig. 2:**
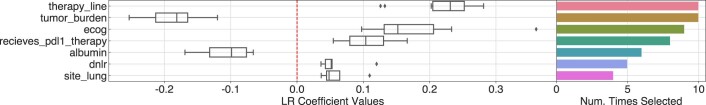
Weights for the LR Clinical model. Coefficient results for clinical features from the 10-fold train–test scheme for the LR Clinical model. The box-and-whisker plots show the mean and the interquartile range (25–75%) as boxes, the minimum and maximum as whiskers up to 1.5 times the IQR and datapoints outside the IQR range as diamonds, for N = 10 fits. The adjoined bar plot shows the number of times, for a given feature, the following criteria were satisfied: (1) if applicable, the feature was selected as part of the radiomics filtering process, (2) the magnitude of the feature’s weight was at least 1 standard deviation from zero and (3) the magnitude of the feature’s weight was greater than 0.01. Only coefficients selected more than once are shown, up to 100. Source data

**Extended Data Fig. 3:**
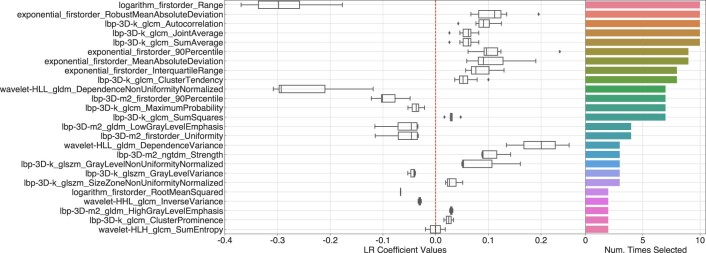
Weights for the LR Rad-PC model. Coefficient results for the parenchymal radiomics features from the 10-fold train–test scheme for the LR Rad-PC model. The box-and-whisker plots show the mean and the interquartile range (25–75%) as boxes, the minimum and maximum as whiskers up to 1.5 times the IQR and datapoints outside the IQR range as diamonds, for N = 10 fits. The adjoined bar plot shows the number of times, for a given feature, the following criteria were satisfied: (1) if applicable, the feature was selected as part of the radiomics filtering process, (2) the magnitude of the feature’s weight was at least 1 standard deviation from zero and (3) the magnitude of the feature’s weight was greater than 0.01. Only coefficients selected more than once are shown, up to 100. Source data

**Extended Data Fig. 4:**
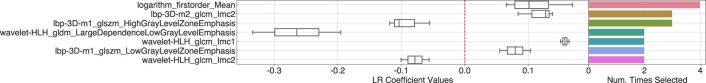
Weights for the LR Rad-LN model. Coefficient results for the lymph node radiomics features from the 10-fold train–test scheme for the LR Rad-LN model. The box-and-whisker plots show the mean and the interquartile range (25–75%) as boxes, the minimum and maximum as whiskers up to 1.5 times the IQR and datapoints outside the IQR range as diamonds, for N = 10 fits. The adjoined bar plot shows the number of times, for a given feature, the following criteria were satisfied: (1) if applicable, the feature was selected as part of the radiomics filtering process, (2) the magnitude of the feature’s weight was at least 1 standard deviation from zero and (3) the magnitude of the feature’s weight was greater than 0.01. Only coefficients selected more than once are shown, up to 100. Source data

**Extended Data Fig. 5:**
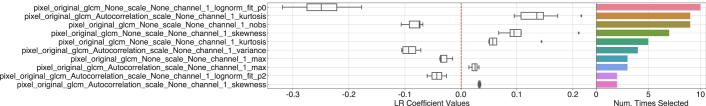
Weights for the LR IHC-A model. Coefficient results for the intensity-based PD-L1 intensity features from the 10-fold train–test scheme for the LR IHC-A model. The box-and-whisker plots show the mean and the interquartile range (25–75%) as boxes, the minimum and maximum as whiskers up to 1.5 times the IQR and datapoints outside the IQR range as diamonds, for N = 10 fits. The adjoined bar plot shows the number of times, for a given feature, the following criteria were satisfied: (1) if applicable, the feature was selected as part of the radiomics filtering process, (2) the magnitude of the feature’s weight was at least 1 standard deviation from zero and (3) the magnitude of the feature’s weight was greater than 0.01. Only coefficients selected more than once are shown, up to 100. Source data

**Extended Data Fig. 6:**
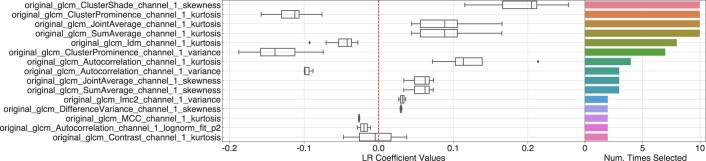
Weights for the LR IHC-G model. Coefficient results for all GLCM-based PD-L1 texture features from the 10-fold train–test scheme for the LR IHC-G model. The box-and-whisker plots show the mean and the interquartile range (25–75%) as boxes, the minimum and maximum as whiskers up to 1.5 times the IQR and datapoints outside the IQR range as diamonds, for N = 10 fits. The adjoined bar plot shows the number of times, for a given feature, the following criteria were satisfied: (1) if applicable, the feature was selected as part of the radiomics filtering process, (2) the magnitude of the feature’s weight was at least 1 standard deviation from zero and (3) the magnitude of the feature’s weight was greater than 0.01. Only coefficients selected more than once are shown, up to 100. Source data

**Extended Data Fig. 7:**
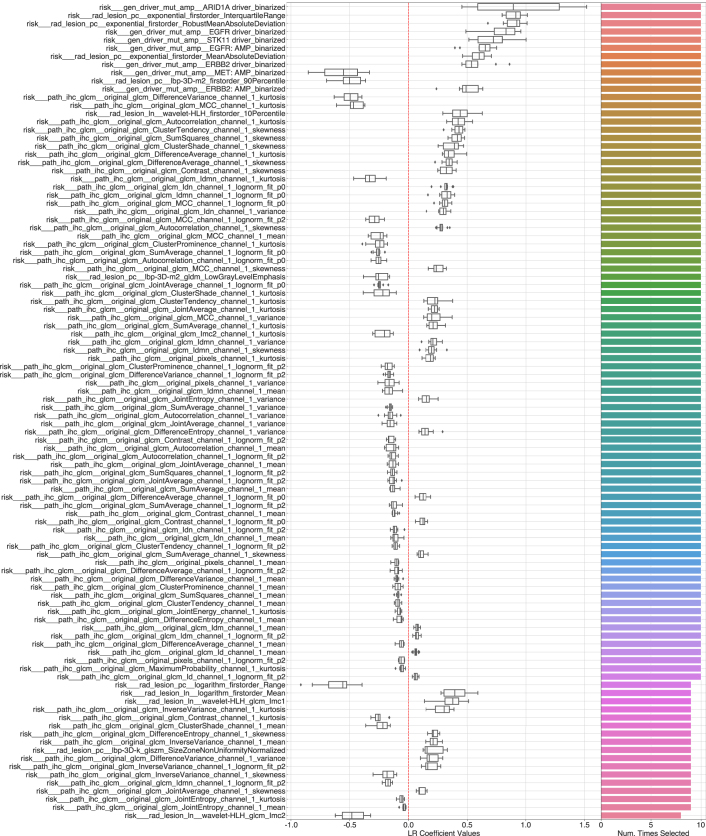
Weights for DyAM Rad+IHC-G + Gen model. Parameter weight results for combined radiomic, GLCM-based PD-L1 texture and genomic features for the DyAM Rad+IHC-G + Gen model. The box-and-whisker plots show the mean and the interquartile range (25–75%) as boxes, the minimum and maximum as whiskers up to 1.5 times the IQR and datapoints outside the IQR range as diamonds, for N = 10 fits. The adjoined bar plot shows the number of times, for a given feature, the following criteria were satisfied: (1) if applicable, the feature was selected as part of the radiomics filtering process, (2) the magnitude of the feature’s weight was at least 1 standard deviation from zero and (3) the magnitude of the feature’s weight was greater than 0.01. Only coefficients selected more than once are shown, up to 100. Source data

**Extended Data Fig. 8:**
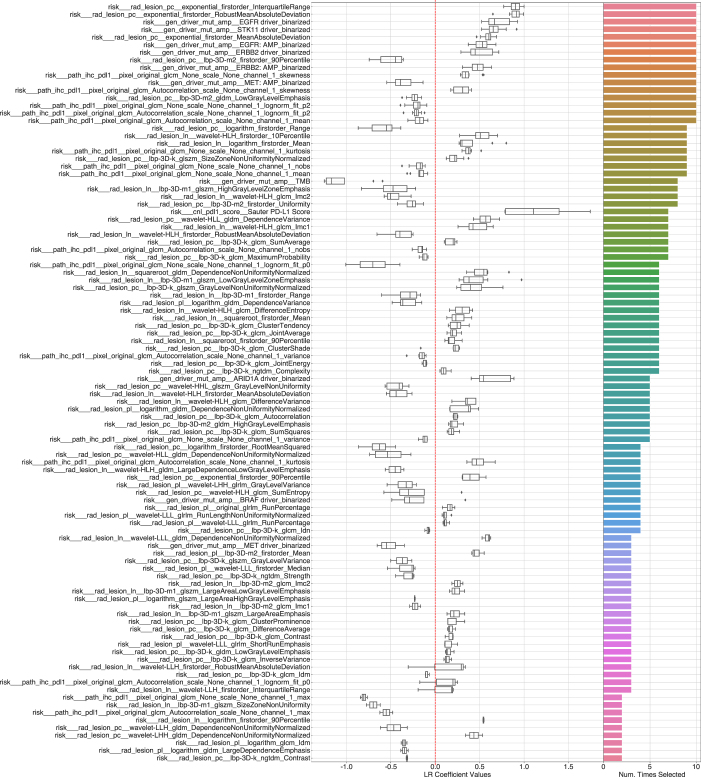
Weights for the DyAM Rad+IHC-A + Gen+PD-L1 model. Parameter weight results for combined radiomic, intensity-based PD-L1 texture, genomic and TPS features for the DyAM Rad+IHC-G + Gen model. The box-and-whisker plots show the mean and the interquartile range (25–75%) as boxes, the minimum and maximum as whiskers up to 1.5 times the IQR and datapoints outside the IQR range as diamonds, for N = 10 fits. The adjoined bar plot shows the number of times, for a given feature, the following criteria were satisfied: (1) if applicable, the feature was selected as part of the radiomics filtering process, (2) the magnitude of the feature’s weight was at least 1 standard deviation from zero and (3) the magnitude of the feature’s weight was greater than 0.01. Only coefficients selected more than once are shown, up to 100. Source data

Having evaluated predictive capacity of unimodal features, we next implemented the DyAM model to evaluate the impact of combining features from radiology, histology and genomics in predicting response to PD-(L)1 blockade (Fig. 5a). The DyAM model outputs include risk attributed to each modality (partial risk score), attention the modality receives (attention weight and share) and the overall score. DyAM has the practical quality of masking modalities in a given patient with no characterization, such as a tumor with negative PD-L1 expression or no segmentable disease in their CT scan. The performance of multimodal integration was assessed using Kaplan–Meier analysis whereby stratification based on multimodal DyAM was more effective at separating high- and low-risk patients than the standard clinical biomarkers of PD-L1 TPS and TMB (Fig. 5b). Using this framework, we then systematically compared unimodal features and various combinations of bimodal and multimodal features (Fig. 5c, F1, precision and recall scores shown in Extended Data Fig. 1, model coefficients are shown in Extended Data Figs. 2–8). In general, layering complementary feature sets improved performance both within and between modalities. For example, DyAM integration of site-specific radiologic features improved prediction from AUC = 0.65, 95% CI 0.57–0.73 to AUC = 0.70, 95% CI 0.62–0.78. Furthermore, a bimodal DyAM model integrating radiological data and PD-L1-derived features (both TPS and IHC texture) resulted in AUC = 0.68 and 95% CI 0.61–0.75, whereas the combination of PD-L1 and genomic features resulted in AUC = 0.72 and 95% CI 0.65–0.79. Combining radiologic and genomic features resulted in the highest bimodal performance (AUC = 0.76, 95% CI 0.69–0.83). Each of these bimodal features improved on either unimodal feature set alone. The best performing, fully automated approach, using three modes of data included IHC-G features, with an AUC = 0.78 (95% CI 0.72–0.85). Finally, using three modes of data with the PD-L1 TPS score resulted in the highest accuracy with AUC = 0.80, 95% CI 0.74–0.86. This was in contrast with averaging the LR scores from all modalities (AUC = 0.72, 95% CI 0.65–0.79). All multimodal DyAM results were significantly higher than null hypothesis AUCs obtained via permutation testing.

**Fig. 6:**
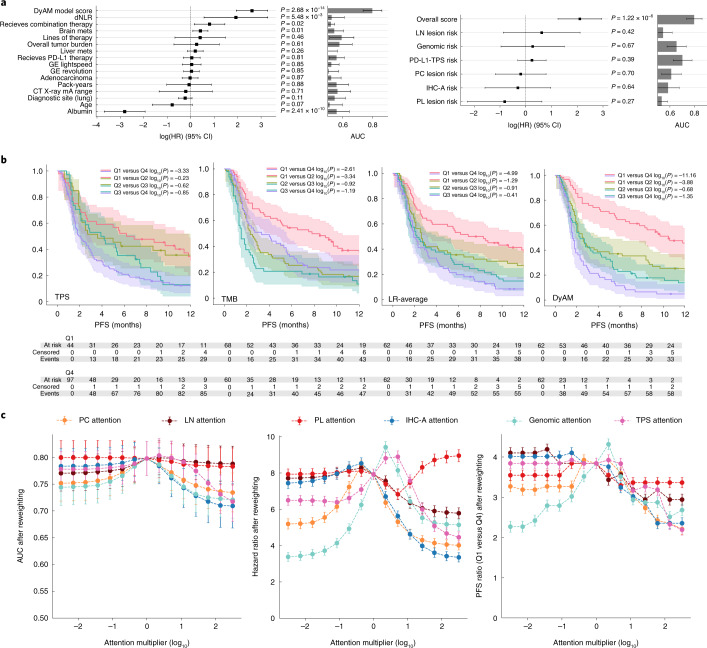
DyAM-based multimodal analysis. **a**, HRs and single feature AUCs of covariates. Overall risk score analysis: forest plot of the DyAM model score with respect to other clinical variables (left). Partial risk score analysis: forest plot of the modality specific risk scores from LR compared to DyAM (right). The vertical dashed lines represent a null HR. *P* values were obtained from the likelihood-ratio test. The bar height and error bar represent the AUC and associated 95% CI based on DeLong’s method^51^ for *n* = 247 patients. **b**, A zoom in of the first 12 months separated by quartile showing separation of early progression events. *P* values were obtained from the log-rank test. **c**, Alpine plots comparing the overall model score after reweighting the input modalities as a function of a multiplier to a single modality’s attention, for the model’s AUC (left), HR (middle) and PFS ratio at 4 months in the lower and higher quartiles (right). For the reweighted AUCs, HRs and PFS ratios, the error bar represents the 68% or 1-sigma CIs based on DeLong’s method^51^, the HR covariance matrix and Poisson standard error on the number of progression events, respectively. Source data

We then compared the DyAM model to established biomarkers of immunotherapy response as well as clinical confounders using multivariable Cox regression (Fig. 6a). The resulting overall score, DyAM risk, was used as input to a multivariable Cox proportional hazards model with derived neutrophil-to lymphocyte ratio (dNLR), pack-years smoking history, age, albumin, tumor burden, presence of brain and liver metastases, tumor histology and scanner parameters. The resulting c-index was 0.74 with several significant features: dNLR (hazard ratio (HR) = 6.87, 95% CI 1.76–26.77, *P* < 0.005), DyAM risk (HR = 13.65, 95% CI 6.97–26.77, *P* < 0.005), albumin (HR = 0.06, 95% CI 0.02–0.14, *P* < 0.005), brain metastasis (HR = 1.51, 95% CI 1.09–2.09 *P* = 0.01) and receiving combination therapy (HR = 2.23 95% CI 1.16–4.29, *P* = 0.02). When comparing the classifier performance against the LR risk scores, only the integrated model was significant (Fig. 6a). We divided the cohort into quartiles using the DyAM score and performed corresponding Kaplan–Meier analysis, focusing on PFS in the first 12 months to highlight the potential of DyAM to separate response groups early after treatment. Progression at 4 months was 21% for the lowest (protective) quartile and 79% for highest (risk) quartile (Fig. 6b), compared to 30% and 60% for the averaging method. Finally, we assessed the effect of reweighting individual data modalities (attention analysis, alpine plots) on the overall model performance (Fig. 6c). In patient subsets with the data modality present, we observed that the removal of lung parenchymal nodule CT texture and genomic alterations had the greatest effect on AUC, whereas the model was robust to the removal of IHC texture and PD-L1 TPS. Non-linear relationships between data modalities indicate an effect of the weighting scheme used within DyAM. At 4 months, the ratio of progression events between the lower and higher quartiles was 3.8 (95% CI 3.7–4.0), which decreases sharply when removing either the CT texture (decreasing to 3.2 (95% CI 3.1–3.3)) or genomic alterations (decreasing to 2.3 (95% CI 2.2–2.4)); however, this early separation did not manifest from either modality in isolation. Model performance decreases for all modes as unimodal attention increases and the DyAM model outperformed simple averaging.

## Discussion

The integration of biomedical imaging, histopathology and genomic assays to guide oncologic decision-making is still in a preliminary phase^28^. Herein, we show that machine-learning approaches that automatically extract discriminative features from disparate modalities result in complementary and combinatorial power to identify high- and low-risk patients with NSCLC who received immunotherapy. Our study represents a proof of principle that information content present in routine diagnostic data, including baseline CT scans, histopathology slides and clinical next-generation sequencing can be combined to improve prognostication for response to PD-(L)1 blockade over any one modality alone and over standard clinical approaches. Integration of these data presents technical difficulty and infrastructure cost; however, our results indicate the potential of integrative approaches. To enable growing interest in deploying data infrastructure to automate the collection, organization and featurization of the data included in this study, the workflows and software are provided for use by the broader community in other cohorts and can be applied beyond NSCLC to other cancers and diseases.

To enable the study, we consulted domain-specific experts to curate features in our dataset. Curation involved segmentation of malignant lesions within CT scans by thoracic radiologists (such as those shown in Fig. 2b) and annotation of digitized PD-L1 IHC slides (such as those used to train the machine-learning classifier to compute the tumor segmentation mask in Fig. 3a) and adjudication of PD-L1 expression by a thoracic pathologist. We also limited genomic and clinical features to those with known associations with NSCLC and immunotherapy outcomes. Heterogeneity of the disparate data modalities presented a unique challenge in their integration, such as, patients with non-segmentable disease or PD-L1-negative tumors, for which PD-L1 expression patterns are not defined. Finally, the most optimal combination of these features is not known and post-fit linear combination or averaging techniques could ignore interactions and correlations between these modalities. The attention gate of DyAM allows for non-linear behavior across the input modalities. The use of attention gating and the generation of partial risk scores has added benefit; it allows for higher-level analysis of multimodal data, such as automatically identifying regions of feature space where certain modalities are more or less predictive. The result was an interpretable, data-driven multimodal prediction model that was also robust to missing data. Reassuringly, the multimodal DyAM model that we derived was not only able to predict short term objective responses better than any modality separately or linearly combined, but also led to enhanced separation of the Kaplan–Meier survival curves that reflected discriminative power within the first few months. This is further evidence that our model could achieve early stratification of true responders from nonresponders, an important criterion for predictive biomarkers and future clinical management decisions. Furthermore, our attention analysis of the DyAM model revealed that all data modalities (radiomics, genomics and pathology) are drivers of this early stratification.

A limitation of our analysis was the size of the multimodal cohort assembled and the restriction to a single center. To ensure consistent training data quality, we chose to include only CT scans that were performed within one institution^32^. Inclusion of CT scans from external institutes warrants further study to investigate the effect of various CT imaging protocols and machine-acquisition parameters on model sensitivity. Digitized PD-L1 IHC slides were similarly chosen from a single center, given differences in staining quality among different laboratories and the use of several different antibodies in clinical practice among institutions^33,34^. Similarly, existing commercial and institution-specific targeted next-generation sequencing panels differ in breadth of coverage and germline filtering techniques, which can introduce challenges for data integration and not all institutions sequence patient-matched healthy specimens to identify germline mutations. These challenges can be mitigated by training models with data from multiple sites to either predict clinical outcomes directly or to perform segmentation in pathological or radiological imaging for downstream analysis; however, a multisite training strategy requires comparable dataset sizes across sites with consistent and rigorous annotations to properly normalize models for heterogeneity and extract robust features. The inclusion of multiple malignancies and imaging modalities could also improve model generalizability, as shown in a multi-institution study involving 1,682 patients by Wu et al. in which robust radiomic signatures were found across three malignancies and two imaging modalities, including one associated with improved survival in immunotherapy-treated NSCLC^35^. Federated learning may provide a principled solution to this challenge; however, its practical use is at very early stages of adoption^36^. Although we were unable to obtain an external validation cohort given the complexity of the data modalities, internal single modality validation cohorts for CT scans and histopathology slides were used as full hold-out sets to validate the findings from the multimodal cohort. Indeed, the models with significant and robust performance in the multimodal cohort showed stable performance in the radiology and pathology cohorts. Despite our best efforts, underperforming models encounter statistical limitations that can be best minimized with further data collection. In addition, we have released all code as open-source software and provide our research-ready datasets for others to reproduce our results, validate the findings at other institutions and ideally extend and further refine our methods.

Incorporation of external data would have also required substantial data assembly, including identification of a cohort of immunotherapy-treated patients with NSCLC with associated RECIST outcomes, CT scans, digitized PD-L1 IHC slides and genomic alterations. Once assembled, scaling and extending our model to incorporate external data would require annotation algorithms to segment CT scan lesions or distinguish tumor from healthy tissue in PD-L1 IHC slides to reduce expert burden. Alternatively, large de-identified datasets from many sites may overcome the need for manual annotation by developing reliable deep-learning models on unannotated data, which can be directly included as part of the DyAM model. In the future, assembly of large well-annotated multi-institutional training datasets will lead to development of robust multimodal classifiers that serve as powerful biomarkers. These decision aids could be integrated into routine clinical care and used to quickly and precisely distinguish responders and nonresponders.

A deeper understanding of features extracted from the data modalities and their relationships to known functional cancer pathways could also aid in feature selection. For example, radiomics characterizations involve the extraction of thousands of features that can be used together to broadly encapsulate intratumoral heterogeneity, but there have been few studies using correlative molecular data to infer functional relationships. This task is further complicated by the fact that many radiomics features are correlated to each other. One study used gene set expression analysis and found an association between radiomics and cell-cycle progression and mitosis^29^. Similarly, correlative molecular data could aid in a more principled selection of features that comprise our PD-L1 IHC texture characterization. Even without molecular data, robust associations between radiological and histological phenotypes, such as those found by Jiang et al., could be a step toward more efficient feature selection^37^.

Other limitations of our analysis include the use of RECIST-derived response end points and the date of the tissue used to assess PD-L1 expression. RECIST outcomes were used instead of directly predicting survival metrics because RECIST criteria are standardized and represent the best proxy in quantifying early treatment response. Additionally, using RECIST outcomes minimize effects from confounders such as indolent disease, future lines of therapy and death unrelated to NSCLC; however, RECIST responses are characterized via measuring tumor size changes on CT scans, which do not fully encompass biological treatment response. With improved biological insight leading to better response metrics, future studies will be needed to refine predictive models. While CT scans are proximal to immunotherapy, PD-L1 expression is often assessed on tissue from the initial diagnosis. This is not ideal as previous treatments can modify the tumor microenvironment, including PD-L1 expression. Our cohort consisted mostly (68%) of patients who received one or more previous lines. While we consider the number of lines of therapy before immunotherapy as a covariate, changes in PD-L1 expression due to previous treatments are not explicitly taken into account.

Our results serve as a proof of principle for the value of multimodal integration. We show that existing data from multiple cancer diagnostic modalities can be annotated, abstracted and combined using computational and machine-learning methods for next-generation biomarker development in NSCLC immunotherapy response prediction. Our DyAM model is a promising approach to integrate multimodal data and future models using larger datasets will make it possible to augment current precision oncology practices in treatment decision-making.

## Methods

This study complies with all relevant ethical regulations and was approved by the institutional review board at MSK Cancer Center (nos. 12-245, 16-1144 and 16-1566). Informed consent was waived as our study was retrospective. Participants were not compensated.

### Data infrastructure to support multimodal data integration

The computational and data infrastructure to support the ingestion, integration and analysis of the multimodal dataset was built through the MSK MIND (Multimodal Integration of Data) initiative. Data pipelines were built to extract and de-identify clinical, radiology, pathology and genomics data from institutional databases. A data lake was built to ingest and manage all data with an on-premise cluster. Workflows were implemented to source the data lake to facilitate analyses using radiology and pathology annotations. All data, metadata and annotation described below were integrated for multimodal analysis.

### Clinical cohorts

The multimodal cohort was formed using the following inclusion criteria: patients with stage IV NSCLC who initiated treatment with anti-PD-(L)1 blockade therapy between 2014 and 2019 at the study institution who had a baseline CT scan, baseline PD-L1 IHC assessment and next-generation sequencing by MSK IMPACT^38^. Patients who received chemotherapy concurrently with immunotherapy were not included. Overall, 247 patients met the inclusion criteria for the training cohort. The multimodal cohort (Table 1) was 54% female with a median age of 68 years (range 38–93 years). Overall, 218 (88%) patients had a history of smoking cigarettes (median 30 pack-years, range 0.25–165). Histological subtypes of NSCLC included 195 (79%) adenocarcinomas, 37 (15%) squamous cell carcinomas, 7 (3%) large cell carcinomas and 8 (3%) NSCLC, NOS (Fig. 1c). Collectively, 169 (68%) patients received one or more lines of therapy before starting PD-(L)1 blockade and 78 (32%) patients received PD-(L)1 blockade as first-line therapy, of which 14 (6%) received therapy in the context of a clinical trial.

The radiology (*n* = 50) validation cohort included patients with a baseline CT which included the chest (± abdomen/pelvis) containing lung lesions >1 cm. The pathology (*n* = 52) validation cohort included patients with a biopsy showing PD-L1-positive (TPS ≥ 1%) NSCLC that was digitized at MSK. Baseline characteristics of the multimodal, radiology and pathology cohorts are shown in Table 1. Best overall response was assessed via RECIST v.1.1 by thoracic radiologists trained in RECIST assessment. Patients who did not progress were censored at the date of last follow-up. PFS was determined from the date of initiating PD-(L)1 blockade therapy until the date of progression or death. OS was determined from the date of initiating PD-(L)1 blockade therapy until the date of death. Those who were still alive were censored at their last date of contact. Clinical, radiologic, pathologic and genomic data were housed in a secure Redcap database.

### CT scans

The baseline CT scan was defined as the closest contrasted scan, including the chest performed within 30 d of starting PD-(L)1 blockade therapy at MSK. Scans were anonymized and quality control was performed to ensure de-identification. Scans were separated into the DICOM format and metadata. All patients underwent multisection CT performed as part of standard clinical care for clinical staging of pulmonary malignancy. CT studies were all performed at our institution (Lightspeed VCT, Discovery CT 750HD; GE Healthcare) and were submitted and uploaded to our picture archiving and communication system.

### Radiological segmentation

Our study was limited to chest imaging to ensure homogeneity of the imaging protocol used. As a result, we only considered chest lesions. Lesion segmentation of primary lung cancers and thoracic metastases was performed manually by three radiologists (N.H. and J.A-F. with 8 years of post-fellowship experience and A.P. with 1 year of post-fellowship experience). Each lesion was segmented by a single radiologist, reviewed by a second and disagreements were resolved with a third. While all radiologists were aware that the patients had lung cancer, they were blinded to patients’ previous treatments and outcomes.

Target lesions were selected in accordance with RECIST v.1.1 criteria (maximum of five target lesions and up to two target lesions per organ). Lesions that were segmented included lung parenchymal, pleural and pathologically enlarged thoracic lymph nodes. Lung and pleural lesions were included when measured as >1.0 cm in the long axis dimension and lymph nodes >1.5 cm in the short axis dimension.

Segmentations were performed on contrast enhanced CTs with 5-mm slices and soft tissue algorithm reconstructions. The segmenting radiologist had access to the clinical text report and positron emission tomography (PET) scan images during segmentation as guides. Lung and soft tissue windows (window level, −600 HU and width, 1,500 HU and window level, 50 HU and width, 350 HU, respectively) were used when appropriate to visually delineate volumes of interest from lung tissue, large vessels, bronchi and atelectasis. Cavitary lesions, lung lesions indistinguishable from surrounding atelectasis and streak artifacts were excluded. Segmented target lesions were categorized and labeled separately by location for textural feature analysis. All segmentations were completed using ITK-SNAP, v.3.4.0 (http://itksnap.org)^39^.

### Radiomics feature analysis

Three thoracic radiologists (N.H., A.P. and J.A-F.) used dictated radiology text reports, PET scan images and RECIST^40^ criteria to guide segmentation. Areas of ambiguity, such as image artifacts from surgical staples, were excluded. A total of 337 lesions from 187 patients, classified into lung parenchymal, pleural and lymph nodes were segmented. The predictive capacity of features extracted from lesions segmented CT scans were analyzed. A variety of radiomics features were computed using all filters available in the pyradiomics Python package v.3.0.1 (ref. ^41^), resulting in 1,688 features. To ease the training of the predictive model, the number of features were reduced by requiring stability with respect to small perturbations of the original segmentation using the method previously used by Zwanenburg et al. to assess robustness of radiomics features^42^. In this method, the original segmentation is perturbed ten times, then radiomic features are computed from each perturbation. We defined a robustness *z* score, which is the ratio of the average inter-lesion variance across the ten perturbations and the feature intra-lesion variance average across the entire multimodal cohort. This value ranges 0–1 and we considered only features with *z* scores less than 0.15. This ensured that, on average, selected features only vary slightly (~15%) across the perturbations with respect to its total dynamic range. The same procedure was implemented in the analysis of the radiology cohort. Investigators were blinded to patient response in the segmentation of the CT scans.

### PD-L1 immunohistochemistry

IHC was performed on 4-μm FFPE tumor tissue sections using a standard PD-L1 antibody (E1L3N; dilution 1:100, Cell Signaling Technologies) validated in the clinical laboratory at the study institution. Staining was performed using an automated immunostaining platform (Bond III, Leica) using heat-based antigen retrieval employing a high pH buffer (epitope retrieval solution-2, Leica) for 30 min. A polymeric secondary kit (Refine, Leica) was used for detection of the primary antibody. Placental tissue served as positive control tissue. Interpretation was performed on all cases by a thoracic pathologist (J.L.S.) trained in the assessment of PD-L1 IHC^43^. Positive staining for PD-L1 in tumor cells was defined as the percent of partial or complete membranous staining among viable tumor cells, known as the TPS. A negative score was defined as staining in <1% of tumor cells or the absence of staining in tumor cells. Slides that did not meet the minimum number of tumor cells for PD-L1 TPS assessment (<100 tumor cells) were not included. The same procedure was implemented to characterize the pathology cohort. Investigators were blinded to patient response in the PD-L1 TPS assessment.

### PD-L1 tissue analysis

PD-L1 IHC-stained diagnostic slides were digitally scanned at a minimum of ×20 magnification for 201 patients using an Aperio Leica Biosystems GT450 v.1.0.0. A deep-learning classifier implemented in the HALO AI software (Indica Labs) was trained to recognize areas of tumor in PD-L1-stained tissue. The training involved annotations across multiple tissue slides to subsequently train the DenseNet AI V2 classifier. The following annotation classes were included: tumor, stroma, lymphocytes, necrosis, fibroelastic scar, muscle, benign lung tissue and glass (absence of tissue). Multiple slides were used to train the classifier to account for site heterogeneity. The trained classifier was then employed across all PD-L1 IHC slides available for the multimodal cohort. Each slide was subsequently manually assessed for tumor segmentation by a thoracic pathologist (J.L.S.) and assigned a specificity score. This score was defined as the proportion of tissue being identified as tumor being correct. Slides with scores below 95% were then manually annotated. Investigators were blinded to patient response in the PD-L1 tissue analysis.

### PD-L1 staining pattern quantification

Once analyzed in the HALO AI software, tumor masks were exported and applied to the original PD-L1 tissue image. The masked tissue image was de-convoluted to separate the PD-L1 IHC from the hematoxylin blue counterstain. GLCMs were used to characterize PD-L1 expression; GLCMs are commonly used in image processing to quantify the similarity of neighboring pixels. Pixel-wise GLCM features were extracted using the pyradiomics Python package. This is performed within pyradiomics by computing GLCM features within a 3 × 3 kernel around each pixel in the image. Summary statistics including mean, s.d. and autocorrelation were computed from the pixel-wise GLCM map for use in downstream analysis.

### Genomics analysis

All patients had panel next-generation sequencing performed on their tumor before the start of treatment. The platform used was the US FDA-authorized MSK-IMPACT platform, which includes somatic mutations, copy number alterations and fusions in 341–468 genes most commonly associated with cancer^31^. Genomic alterations in genes commonly associated with NSCLC and thought to associate with anti-PD-L1 therapy response were extracted from cBioPortal^44,45^. This included altered oncogenes (*EGFR*, *ALK*, *ROS1*, *RET*, *HER2/ERBB2*, *BRAF* and *MET*), altered tumor suppressors (*STK11*) and altered transcription regulators (*ARID1A*). All alterations were annotated by OncoKB^46^ to determine whether they are potentially oncogenic/driver events. TMB, a measure of the number of somatic mutations per megabase of the interrogated genome, is a US FDA-approved predictive biomarker of immunotherapy response in NSCLC^47^. TMB was calculated based on all identified mutations divided by the coding region captured in the MSK-IMPACT panels. Multivariable analysis was conducted using Cox proportional hazards modeling adjusting for the genes described as well as TMB.

### Logistic regression

LR with elasticnet penalty was used to predict binary outcomes given feature vectors. We used the implementation provided in sklearn v.0.24.0 using the saga optimizer, *C* = 0.1, a l1-ratio of 0.5 and balanced class weights.

### Multiple-instance logistic regression

Multiple-instance learning is a class of machine learning where sets of training data (bags) share a common label^48^. Attention-based pooling as developed by Ilse et al.^49^ extended the multiple-instance learning paradigm to assign attention-based weights to each instance, which are a function of the instance features and optimized for prediction of an outcome. This technique is designed for an unfixed number of input instances with fixed feature size, such as the 1–5 texture feature vectors for each lesion encountered in the radiology analysis. The final output score is a weighted sum of the same LR model applied for each lesion, where the weights are dynamically determined. In the single-lesion case, we were able to recover similar results as compared to the standard LR. This technique treats each instance equally as there is a single set of parameters shared across all instances.

The model was developed and fit using pytorch v.1.8.0, with a hidden size of 32, balanced class weights, binary cross-entropy loss, 0.005 learning rate, 250 steps and L2 regularization of 0.005 using the Adam optimizer.

### Multimodal dynamic attention with masking

Multiple-instance learning typically involves a single group or bag, of homogenous instances; however, in the multimodal case we encounter heterogenous instances. Furthermore, even though the textures derived from each lesion are the same shape, lesions in different sites may map to the predictor in different ways, such that a shared set of parameters may not be optimal. We thus encounter a MIL paradigm where we have a fixed-sized bag of heterogeneous instances or modes. For this study, the modes of data utilized were one-dimensional feature vectors derived from PD-L1 IHC performed on diagnostic tissue, segmented radiological CT scans delineated by lesion site and genomic alterations. We utilized the same dynamic pooling scheme in our model. Each input mode has its own set of trainable parameters: one to map the input vector to the label (risk parameters) and one to map the input vector to an attention score (attention parameters), which competes with the other input modes in a similar way to the standard multiple-instance learning model. As attention-based weights are trainable, the model learns which input modes are most relevant to treatment response. In contrast to multiple-instance learning, the final output score is a weighted sum of individually optimized LR models applied for each modality; this technique treats each mode specifically, while still optimizing all parameters in concert. Finally, as zero attention corresponds to a modality having no correspondence to the predictor, this model was expanded by adding a masking function that sets any missing mode’s attention to zero to address missing data (such as non-segmentable disease in CT). We call this the DyAM model.

The model was developed and fit using pytorch v.1.8.0, with balanced class weights, binary cross-entropy loss, 0.01 learning rate, 125 steps and L2 regularization strength of 0.001 using the Adam optimizer. When only one input modality was present, the attention gate was disabled to reduce complexity.

### Attention analysis

Given the normalized attention weights and risk scores per modality, one can reweight the attention layer to assess how an increase or decrease in a modality’s attention impacts the model’s performance. This can be performed schematically by multiplying the attention vector $\bar{a}$ by a multiple of the *k*th unit vector $m\hat{e}_k$, taking the dot product with the risk vector $\bar{r}$ and re-normalizing the score.

$$score = \left(m\hat{e}_k \, \circ \, \bar{a}\right) \cdot \bar{r}/\Sigma\left(m\hat{e}_k \, \circ \, \bar{a}\right)$$

The new weights can be determined as follows:

$$a_{i=k} = ma_k/(1 + (m-1)a_k),a_{i\neq k} = a_i/(1+(m-1)a_k)$$

If *a*_*k*_ = 0.5 and *m* = 10, the new attention of the *k*th modality increases to 0.91 and the other modalities’ attention weights decrease by a factor of 0.18. Conversely, at *m* = 0.01, the attention of the *k*th modality decreases to 0.01 and the other modalities’ attention weights increase by a factor of 2. At high multiples, the model approaches a unimodal model dominated by that modality. At low multiples, the model approaches a *N*-1 model with that modality dropped out. We scanned *m* in the *log*_10_ scale from −2 to 2 and recomputed performance metrics (AUC, HR and PFS ratio) at each step.

### Statistical analysis

**Extended Data Fig. 9:**
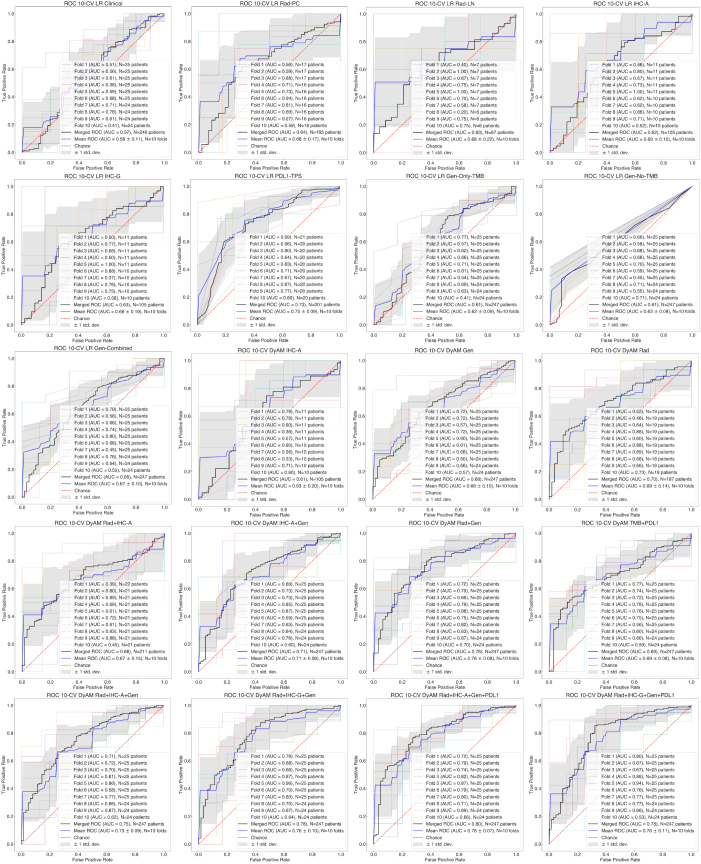
Receiver operating characteristics for all models using the 10-fold train–test breakdown. ROC curves showing the results from individual folds as thin colored lines, the averaged and ±1 standard deviation ROC curve calculated from the distribution of the true positive rate in bins of the false positive rate across all folds as a blue curve surrounded by a gray band and the merged AUC as a black curve. For each model and fold result, the subset of N = 247 patients included depending on the available modalities and train–test split is indicated. Source data

**Extended Data Fig. 10:**
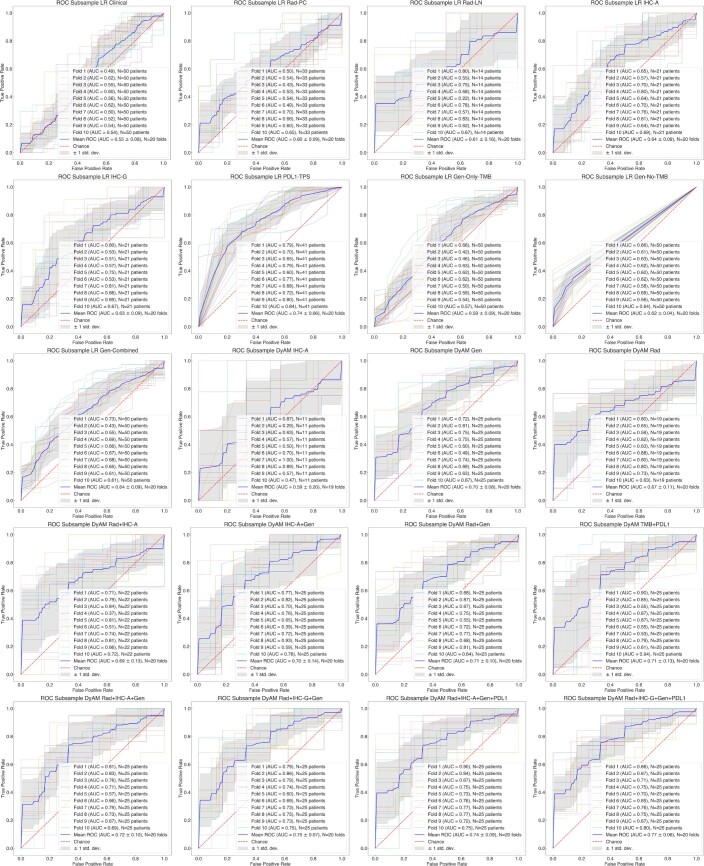
Receiver operating characteristics for all models using repeated subsampling. ROC curves showing the results from the first 10 subsamples as thin colored lines and the averaged and ±1 standard deviation ROC curve calculated from the distribution of the true positive rate in bins of the false positive rate across all subsamples as a blue curve surrounded by a gray band. For each model and fold result, the subset of N = 247 patients included depending on the available modalities and train–test split is indicated. Source data

There were no formal sample size calculations performed in advance. The multimodal cohort was selected based on the existing number of patients at MSK who met all inclusion criteria. Group comparisons were performed using two-sided Mann–Whitney *U*-tests. AUC was used as a measure of performance of a biomarker on predicting response versus nonresponse. We performed tenfold cross-validation where scores from the validation folds were combined to form predictions across the entire multimodal cohort. Patients were randomly sorted into the folds. All feature selection and feature rescaling was performed on the training fold in each iteration of the cross-validation analysis. The binary cutoff used to determine the predicted response was determined for each patient using only training data. Hyperparameters were established from the first fold and applied consistently for each model. To explicitly evaluate the sensitivity of model fitting to the data used for training, we also performed a subsampling analysis where the data was randomly sampled 100 times using 90% for training and the remaining 10% for testing. All models were trained in an end-to-end manner with corresponding cross-validation across folds (Extended Data Fig. 9) and subsampling (Extended Data Fig. 10). We also report the F1-score, precision and recall for all models in Supplementary Table 1 and Extended Data Fig. 1. Model coefficients are shown in Extended Data Figs. 2–8. A multivariable Cox proportional hazards model was developed with the Lifelines Python package v.0.25.7 (ref. ^50^) to compare the performance of this model to existing biomarkers and calculate adjusted HRs. The Lifelines package was used to compute survival curves estimated using the Kaplan–Meier method. All statistical tests were two-sided with a significance level of 2.5% for each tail. Assessment of classifier performance was performed consistently for each analysis, using AUCs computed across the entire multimodal cohort by merging classifier scores obtained in the validation folds of tenfold cross-validation. Model outputs were calibrated to an optimal threshold using the Youden index and rescaled to a unit variance. The 95% CIs on these AUCs were calculated using DeLong’s methods for computing the covariance of unadjusted AUCs^51^. Violins were smoothed using the default Gaussian kernel density estimation in seaborn v.0.11.1. Significance of the AUCs were obtained against the hypothesis of meaningless class labels using perturbation testing with 20 iterations with the covariance error and s.e.m. propagated in quadrature.

### Reporting summary

Further information on research design is available in the Nature Research Reporting Summary linked to this article.

## Supplementary information

Supplementary Information Supplementary Table 1

Reporting Summary

## Data Availability

All data are publicly available at synapse: https://www.synapse.org/#!Synapse:syn26642505. The genomic features of the cohort can be explored on cbioportal: https://www.cbioportal.org/study/summary?id=lung_msk_mind_2020. Source data have been provided as Source Data files. Source data are provided with this paper.

## Source data

Source Data Fig. 1 Source clinical data and distributions.
Source Data Fig. 2 First and second PCA decomposition components and associated site, response, radiology model AUC results and 95% CI.
Source Data Fig. 3 Raw GLCM calculations and pathology model AUC results.
Source Data Fig. 4 Cox proportional hazard model statistical results, fit coefficients and genomic model AUC results.
Source Data Fig. 5 Raw time-series analysis event data and all summary AUC results.
Source Data Fig. 6 Cox proportional hazard model statistical results, raw time-series analysis event data and source data for alpine results.
Source Data Extended Data Fig. 1 Source metric scores for all models.
Source Data Extended Data Fig. 2 Raw coefficient or feature weight values.
Source Data Extended Data Fig. 3 Raw coefficient or feature weight values.
Source Data Extended Data Fig. 4 Raw coefficient or feature weight values.
Source Data Extended Data Fig. 5 Raw coefficient or feature weight values.
Source Data Extended Data Fig. 6 Raw coefficient or feature weight values.
Source Data Extended Data Fig. 7 Raw coefficient or feature weight values.
Source Data Extended Data Fig. 8 Raw coefficient or feature weight values.
Source Data Extended Data Fig. 9 All model scores and labels for each patient using the tenfold train–test scheme.
Source Data Extended Data Fig. 10 All model scores and labels for each patient using the repeated subsampling scheme.

## Code availability

Feature processing and engineering code is available as part of Luna: https://github.com/msk-mind/luna/
