# Supplementary material for: Multimodal integration of radiology, pathology and genomics for prediction of response to PD-(L)1 blockade in patients with non-small cell lung cancer
Source: Nat Cancer. 2022 Aug 29;3(10):1151–64. doi: 10.1038/s43018-022-00416-8 (PMC9586871; doi:10.1038/s43018-022-00416-8)
Supplement: Supplementary file 1 — Supplementary Table 1 [file 43018_2022_416_MOESM1_ESM.pdf]

nature portfolio

https://doi.org/10.1038/s43018-022-00416-8

Supplementary information

Multimodal integration of radiology, pathology and genomics for prediction of response to PD-(L)1 blockade in patients with non-small cell lung cancer

In the format provided by the authors and unedited

Model	F1-score	Precision Score	Recall Score	AUC	Accuracy
LR Clinical	0.680982	0.787234	0.600000	0.569606	0.577236
LR Rad-PC	0.736364	0.810000	0.675000	0.639341	0.644172
LR Rad-LN	0.682927	0.717949	0.651163	0.626938	0.611940
LR Rad-Average	0.722892	0.796460	0.661765	0.645040	0.631016
MILR Rad-Lesions	0.632479	0.755102	0.544118	0.620314	0.540107
LR IHC-A	0.666667	0.701754	0.634921	0.623961	0.619048
LR IHC-G	0.666667	0.722222	0.619048	0.633787	0.628571
LR PDL1-TPS	0.788530	0.839695	0.743243	0.729284	0.706468
LR Path-A-Average	0.802817	0.838235	0.770270	0.695117	0.721393
LR Path-G-Average	0.811189	0.840580	0.783784	0.694225	0.731343
LR Gen-Only-TMB	0.794444	0.817143	0.772973	0.606931	0.700405
LR Gen-No-TMB	0.486056	0.924242	0.329730	0.605100	0.477733
LR Gen-Average	0.769231	0.813253	0.729730	0.658195	0.672065
LR Gen-Combined	0.769231	0.813253	0.729730	0.654795	0.672065
DyAM IHC-A	0.655462	0.696429	0.619048	0.605631	0.609524
DyAM Gen	0.716511	0.845588	0.621622	0.675850	0.631579
DyAM Rad	0.773946	0.808000	0.742647	0.701413	0.684492
DyAM Rad+IHC-A	0.755245	0.812030	0.705882	0.680753	0.668246
DyAM IHC-A+Gen	0.777448	0.861842	0.708108	0.714996	0.696356
DyAM Rad+Gen	0.785924	0.858974	0.724324	0.759285	0.704453
DyAM TMB+PDL1	0.746269	0.833333	0.675676	0.693112	0.655870
DyAM Rad+IHC-A+Gen	0.783626	0.853503	0.724324	0.754228	0.700405
DyAM Rad+IHC-G+Gen	0.794118	0.870968	0.729730	0.782563	0.716599
DyAM Rad+IHC-A+Gen+PDL1	0.818713	0.891720	0.756757	0.798169	0.748988
DyAM Rad+IHC-G+Gen+PDL1	0.836676	0.890244	0.789189	0.783348	0.769231
LR Multimodal-Average	0.777778	0.847134	0.718919	0.723365	0.692308
