# Supplementary material for: Multimodal integration of radiology, pathology and genomics for prediction of response to PD-(L)1 blockade in patients with non-small cell lung cancer
Source: Nat Cancer. 2022 Aug 29;3(10):1151–64. doi: 10.1038/s43018-022-00416-8 (PMC9586871; doi:10.1038/s43018-022-00416-8)
Supplement: Supplementary file 2 — Reporting Summary [file 43018_2022_416_MOESM2_ESM.pdf]

nature portfolio

Corresponding author(s): Sohrab Shah

Last updated by author(s): May 26, 2022

Reporting Summary

Nature Portfolio wishes to improve the reproducibility of the work that we publish. This form provides structure for consistency and transparency in reporting. For further information on Nature Portfolio policies, see our Editorial Policies and the Editorial Policy Checklist.

Statistics

For all statistical analyses, confirm that the following items are present in the figure legend, table legend, main text, or Methods section.

n/a	Confirmed	
☐	☒	The exact sample size (*n*) for each experimental group/condition, given as a discrete number and unit of measurement
☒	☐	A statement on whether measurements were taken from distinct samples or whether the same sample was measured repeatedly
☐	☒	The statistical test(s) used AND whether they are one- or two-sided<br>*Only common tests should be described solely by name; describe more complex techniques in the Methods section.*
☐	☒	A description of all covariates tested
☒	☐	A description of any assumptions or corrections, such as tests of normality and adjustment for multiple comparisons
☐	☒	A full description of the statistical parameters including central tendency (e.g. means) or other basic estimates (e.g. regression coefficient) AND variation (e.g. standard deviation) or associated estimates of uncertainty (e.g. confidence intervals)
☐	☒	For null hypothesis testing, the test statistic (e.g. *F*, *t*, *r*) with confidence intervals, effect sizes, degrees of freedom and *P* value noted<br>*Give P values as exact values whenever suitable.*
☒	☐	For Bayesian analysis, information on the choice of priors and Markov chain Monte Carlo settings
☒	☐	For hierarchical and complex designs, identification of the appropriate level for tests and full reporting of outcomes
☒	☐	Estimates of effect sizes (e.g. Cohen's *d*, Pearson's *r*), indicating how they were calculated

Our web collection on statistics for biologists contains articles on many of the points above.

Software and code

Policy information about availability of computer code

Data collection	Electronic health records were used to acquire clinical outcomes, and PACS was used to review CT scans for quality. cBioPortal was used to acquire genomic information.
Data analysis	All custom code: https://www.synapse.org/#!Synapse:syn26642505<br>Feature processing and engineering: https://github.com/msk-mind/luna<br>Radiological annotation: ITK-SNAP v3.4.0<br>Pathological annotation: HALO v3.2.1851<br>Survival analysis: lifelines 0.25.7 python package<br>Radiomics analysis: pyradiomics 3.0.1 python package<br>Logistic modeling: scikit-learn v0.24.0 python package

For manuscripts utilizing custom algorithms or software that are central to the research but not yet described in published literature, software must be made available to editors and reviewers. We strongly encourage code deposition in a community repository (e.g. GitHub). See the Nature Portfolio guidelines for submitting code & software for further information.

Data

Policy information about availability of data

All manuscripts must include a data availability statement. This statement should provide the following information, where applicable:

- Accession codes, unique identifiers, or web links for publicly available datasets
- A description of any restrictions on data availability
- For clinical datasets or third party data, please ensure that the statement adheres to our policy

Radiology, pathology and genomic data: https://www.synapse.org/#!Synapse:syn26642505
cbioportal link: https://www.cbioportal.org/study/summary?id=lung_msk_mind_2020

Field-specific reporting

Please select the one below that is the best fit for your research. If you are not sure, read the appropriate sections before making your selection.

☒ Life sciences ☐ Behavioural & social sciences ☐ Ecological, evolutionary & environmental sciences

For a reference copy of the document with all sections, see nature.com/documents/nr-reporting-summary-flat.pdf

Life sciences study design

All studies must disclose on these points even when the disclosure is negative.

Sample size	No prior sample size calculation was performed. The sample size of 247 was chosen based overlap of available data modalities (CT imaging, pre-treatment PD-L1 biopsy tissue and genomics) and the requirement that the patient present Stage IV non-small cell lung cancer treated with anti-PD-L1 checkpoint blockade between 2014-2019.
Data exclusions	All patients who met the cohort definition criteria were included. The inclusion criteria was established before the analysis was performed.
Replication	Since we used data collected during routine clinical care, all data was retrospective and replication was not possible.
Randomization	We used 10X cross validation to divide the patients into 10 random groups which were collectively used to assess classifier performance.
Blinding	Clinical outcomes were blinded to those doing radiological and pathological annotation.

Reporting for specific materials, systems and methods

We require information from authors about some types of materials, experimental systems and methods used in many studies. Here, indicate whether each material, system or method listed is relevant to your study. If you are not sure if a list item applies to your research, read the appropriate section before selecting a response.

Materials & experimental systems

n/a	Involved in the study	
☐	☒	Antibodies
☒	☐	Eukaryotic cell lines
☒	☐	Palaeontology and archaeology
☒	☐	Animals and other organisms
☐	☒	Human research participants
☒	☐	Clinical data
☒	☐	Dual use research of concern

Methods

n/a	Involved in the study	
☒	☐	ChIP-seq
☒	☐	Flow cytometry
☒	☐	MRI-based neuroimaging

Antibodies

Antibodies used	IHC was performed on 4 µm FFPE tumor tissue sections using a standard PD-L1 antibody (E1L3N; dilution 1:100, Cell Signaling Technologies, Danvers, MA).
Validation	The PD-L1 antibody was validated at the study institution according to manufacturer instructions.

Human research participants

Policy information about studies involving human research participants

Population characteristics	Patients were selected based on the following inclusion criteria: patients with stage IV NSCLC who initiated treatment with anti-PD-(L)1 blockade therapy between 2014-2019 at the study institution who had a baseline CT scan, baseline PD-L1 IHC assessment and next generation sequencing by MSK-IMPACT. Patients who received chemotherapy concurrently with immunotherapy were not included. 247 patients met inclusion criteria for the training cohort. The multimodal cohort was 54% female with median age of 68 years (range = 38-93 years). Overall, 218 (88%) patients had a history of smoking cigarettes (median 30 pack-years, range 0.25-165). Histological subtypes of NSCLC included 195 (79%) adenocarcinomas, 37 (15%) squamous cell carcinomas, 7 (3%) large cell carcinoma, and 8 (3%) NSCLC, not otherwise specified (NOS) . Collectively, 169 (68%) patients received one or more lines of therapy prior to starting PD-(L)1 blockade, while 78 (32%) patients received PD-(L)1 blockade as first line therapy, of which 14 (6%) received therapy in the context of a clinical trial. Cohort characteristics are also shown in Table 1.
Recruitment	All patients who met inclusion criteria were included in the cohort.
Ethics oversight	This study was approved by the institutional review board at Memorial Sloan Kettering Cancer Center (IRB #12-245, 16-1144, and 16-1566). Informed consent was waived as our study was retrospective.

Note that full information on the approval of the study protocol must also be provided in the manuscript.
